# Immunohistochemical analysis of adipokine and adipokine receptor expression in the breast tumor microenvironment: associations of lower leptin receptor expression with estrogen receptor-negative status and triple-negative subtype

**DOI:** 10.1186/s13058-020-1256-3

**Published:** 2020-02-11

**Authors:** Adana A. M. Llanos, Yong Lin, Wenjin Chen, Song Yao, Jorden Norin, Marina A. Chekmareva, Coral Omene, Lei Cong, Angela R. Omilian, Thaer Khoury, Chi-Chen Hong, Shridar Ganesan, David J. Foran, Michael Higgins, Christine B. Ambrosone, Elisa V. Bandera, Kitaw Demissie

**Affiliations:** 1 Department of Biostatistics and Epidemiology, Rutgers School of Public Health, Piscataway, NJ USA; 2 Rutgers Cancer Institute of New Jersey, New Brunswick, NJ USA; 3 Department of Pathology and Laboratory Medicine, Robert Wood Johnson Medical School, New Brunswick, NJ USA; 4 Department of Cancer Prevention and Control, Roswell Park Comprehensive Cancer Center, Buffalo, NY USA; 5 Department of Cell Biology and Neuroscience, Rutgers School of Arts and Sciences, New Brunswick, NJ USA; 6 Department of Medicine, Robert Wood Johnson Medical School, New Brunswick, NJ USA; 7 Department of Pharmacology, Robert Wood Johnson Medical School, New Brunswick, NJ USA; 8 Department of Molecular and Cellular Biology, Roswell Park Comprehensive Cancer Center, Buffalo, NY USA; 9 Department of Epidemiology and Biostatistics, SUNY Downstate Health Sciences University School of Public Health, Brooklyn, NY USA

**Keywords:** Leptin, Leptin receptor, Adiponectin, Adiponectin receptors 1 and 2, IHC expression, Breast cancer clinicopathology, Aggressive tumor features

## Abstract

**Background:**

The molecular mechanisms underlying the association between increased adiposity and aggressive breast cancer phenotypes remain unclear, but likely involve the adipokines, leptin (LEP) and adiponectin (ADIPOQ), and their receptors (LEPR, ADIPOR1, ADIPOR2).

**Methods:**

We used immunohistochemistry (IHC) to assess LEP, LEPR, ADIPOQ, ADIPOR1, and ADIPOR2 expression in breast tumor tissue microarrays among a sample of 720 women recently diagnosed with breast cancer (540 of whom self-identified as Black). We scored IHC expression quantitatively, using digital pathology analysis. We abstracted data on tumor grade, tumor size, tumor stage, lymph node status, Ki67, estrogen receptor (ER), progesterone receptor (PR), and human epidermal growth factor receptor 2 (HER2) from pathology records, and used ER, PR, and HER2 expression data to classify breast cancer subtype. We used multivariable mixed effects models to estimate associations of IHC expression with tumor clinicopathology, in the overall sample and separately among Blacks.

**Results:**

Larger proportions of Black than White women were overweight or obese and had more aggressive tumor features. Older age, Black race, postmenopausal status, and higher body mass index were associated with higher LEPR IHC expression. In multivariable models, lower LEPR IHC expression was associated with ER-negative status and triple-negative subtype (*P* < 0.0001) in the overall sample and among Black women only. LEP, ADIPOQ, ADIPOR1, and ADIPOR2 IHC expression were not significantly associated with breast tumor clinicopathology.

**Conclusions:**

Lower LEPR IHC expression within the breast tumor microenvironment might contribute mechanistically to inter-individual variation in aggressive breast cancer clinicopathology, particularly ER-negative status and triple-negative subtype.

## Background

In the United States (US), Black women have higher breast cancer incidence at a younger age (e.g., ≤ 45 years) and are more frequently diagnosed with cancers exhibiting more aggressive phenotypes compared to their White counterparts [1–3]. US cancer incidence data during 2008 and 2012 show that Black women have the lowest incidence of breast cancers characterized as hormone receptor-positive (positive for estrogen receptor [ER] and/or progesterone receptor [PR]) and human epidermal growth factor receptor 2 (HER2)-negative and the highest incidence of hormone receptor-negative and HER2-negative breast cancers when compared to other racial/ethnic groups [1]. Furthermore, breast cancer mortality among Black women continues to be higher than among White women by at least 41% [1, 3]. Given that Black women also tend to have higher age-adjusted prevalence rates of obesity (approximately 57% among non-Hispanic Black vs. 40% among non-Hispanic White women [4]), we hypothesize that inter-individual variation in adiposity and adiposity-related biomarkers (namely leptin [LEP], leptin receptor [LEPR], adiponectin [ADIPOQ], adiponectin receptor 1 [ADIPOR1], and adiponectin receptor 2 [ADIPOR2]) within the breast tumor microenvironment might be involved in the observed racial differences in aggressive breast cancer phenotypes between Black and White women.

Increased levels of adiposity are consistently associated with elevated risk of postmenopausal breast cancer [5, 6] and poorer breast cancer outcomes, irrespective of menopausal status [6–8]. However, the molecular mechanisms underlying these associations are not well understood. The relationship between adiposity and breast cancer might be explained partly by biological effects of the adipokines, ADIPOQ, and LEP, which are secreted by adipocytes [9–14]. Epidemiologic data have shown an inverse association of circulating ADIPOQ levels with increasing body mass index (BMI) [15–17] and breast cancer risk [18–21]. Conversely, circulating LEP levels are positively associated with BMI [22, 23] and also breast cancer risk in some studies [18, 24–26].

ADIPOQ is the most abundantly produced hormone in adipose tissue [16, 27] and, with its receptors (ADIPOR1 and ADIPOR2), is expressed in both histologically normal and malignant breast tissues [28, 29]. ADIPOQ has anti-inflammatory and anti-atherogenic properties [27, 30], and is known to inhibit cellular proliferation and to promote apoptosis [11, 14, 31], implying a protective role in breast carcinogenesis and progression. ADIPOQ expression is downregulated by elevated adiposity, glucocorticoids, β-adrenergic agonists, and TNF-α, and upregulated by leanness, cold exposure, adrenalectomy, and IGF-1 [32, 33]. LEP, also produced in adipose tissues, is similarly expressed in histologically normal and malignant breast tissues, as is the LEPR [34, 35]. LEP is a key growth factor and may be involved in breast carcinogenesis and progression by promoting cell growth, proliferation, and angiogenesis [36–39]. Once bound to LEPR, LEP induces the activation of several signaling pathways (including Janus kinase-signal transducer and activator of transcription [JAK/STAT], mitogen-activated protein kinase [MAPK], insulin receptor substrate 1 [IRS1], and suppressor of cytokine signaling 3 [SOCS3] [9, 36, 40]) that modulate cellular proliferation and survival [9], and these activities have been demonstrated in breast epithelial cells [36, 40]. In summary, ADIPOQ and LEP act on breast epithelial tissues through endocrine pathways as well as locally through autocrine and/or paracrine pathways [28, 41], and, along with their receptors, likely contribute to breast carcinogenesis, progression, and aggressiveness through these mechanisms.

The objective of this study was to determine whether immunohistochemical (IHC) expression of LEP, LEPR, ADIPOQ, ADIPOR1, and ADIPOR2 are associated with breast tumor clinicopathological characteristics, namely those that are indicative of aggressive phenotypes among Black and White women newly diagnosed with breast cancer, including poor differentiation (higher tumor grade), larger tumor size, positive lymph node status, unfavorable Ki67 status (Ki67+), ER− status, HER2+ status, and non-luminal HER2-enriched (HER2-E) and triple-negative (TN) subtypes.

## Methods

### Study sample and data collection

In this study, we conducted a case-only analysis that included 720 incident, primary invasive breast cancer cases diagnosed from 2001 to 2015 and enrolled in the Women’s Circle of Health Study (WCHS). The study design of WCHS is described elsewhere [42]. Briefly, newly diagnosed breast cancer cases with histologically confirmed ductal carcinoma in situ (DCIS, stage 0) or invasive breast cancer (stages I–IV), who self-identified as either Black/African American or White/European American, were 20–75 years of age, able to complete an interview in English, and had no history of cancer except non-melanoma skin cancer, were eligible to participate. Data collection for the WCHS was conducted through in-person assessments and included interviewer-administered questionnaires as well as anthropometric and body composition measurements [43]. The baseline interview ascertained information on sociodemographics, as well as established or probable breast cancer risk factors, including family and personal health history, reproductive history, hormone therapy use, and lifestyle exposures. Anthropometric measurements (height, weight, waist and hip circumference measures) and body composition measures (lean and fat mass, percent body fat) were also taken at the in-person, baseline interview using standardized protocols and instruments [43].

Upon consent for medical records release, medical and pathology records from all providers and institutions where WCHS participants reported receiving breast cancer care were retrieved [44]. Trained abstractors reviewed and abstracted relevant data from each record and entered data into an electronic database [44]. For quality assurance, values were checked for errors during data entry, and if errors were detected, the original abstractor was contacted with instructions to re-check the medical records/pathology report, allowing for confirmation of the recorded data. For the present analysis, we utilized abstracted data on tumor clinicopathology. Tumor grade was defined as grades 1 through 3: grade 1 denoted well-differentiated tumors, grade 2 denoted moderately differentiated tumors, and grade 3 denoted poorly differentiated tumors. Tumor size (cm) was classified into three categories: < 1.0 cm, 1.0–2.0 cm, and > 2.0 cm. American Joint Committee on Cancer (AJCC) stage data was recorded as stages 0 through IV; we considered four categories in the main analysis, stage 0, I, II, and a combined category that included stages III and IV. Lymph node status was defined as node negative or node positive, based on the presence of cancer cells in axillary lymph nodes. Ki67 staining was classified as Ki67+/unfavorable, Ki67 borderline, or Ki67−/favorable (due to the lack of abstraction of clinically relevant percentages of Ki67 staining). Ki67 status was then dichotomized where cases coded as Ki67−/favorable or borderline were classified as having “favorable” Ki67 status, while cases coded as Ki67+ were classified as having “unfavorable” Ki67 status. We used surrogate classifications of ER status, PR status, and HER2 status, which were based on IHC expression of ER and PR, and overexpression of HER2 (by IHC and/or fluorescence in situ hybridization [FISH]). Using these classifications, we approximated breast cancer subtype into four mutually exclusive, clinically recognized subtypes: luminal A (ER+ and/or PR+/HER2−), luminal B (ER+ and/or PR+/HER2+), HER2-E (ER−/PR−/HER2+), and TN (ER−/PR−/HER2−).

This study was approved by the Institutional Review Boards of all participating institutions, and all study participants provided written informed consent prior to study enrollment.

### Collection of archived breast tumor specimens and tissue microarray construction

In the WCHS, tumor blocks and/or slides were collected from hospitals after written consent was obtained from study participants. The retrieval rate of archived breast tumor specimens was approximately 85% in the study. Upon receipt at the Data Bank and BioRepository (DBBR) at Roswell Park Comprehensive Cancer Center, a study pathologist (TK) reviewed the hematoxylin and eosin (H&E) slides and circled areas where cores were taken for tissue microarrays (TMAs). TMA cores ranged in size from 0.6 to 1.2 μm in diameter, and most WCHS participants’ tumors were represented by at least two TMA cores (range, 1 to 6 cores), which were placed into a recipient formalin-fixed paraffin-embedded (FFPE) block. The location of each core was recorded in a detailed TMA map file. The completed TMAs were stored at room temperature.

### Immunohistochemistry

All IHC staining was performed using Ventana Discovery XT Automated Slide Stainer (Ventana Medical Systems, Inc., Tucson, AZ, USA). Deparaffinization, antigen retrieval, blocking, DAB detection, counterstain, post-counterstain, and slide cleaning steps were automated on the Discovery XT. Primary antibodies and secondary antibodies were manually applied at programmed steps. The following primary antibodies were used: rabbit monoclonal OB (leptin) antibody (1:40 dilution; Santa Cruz, cat #sc-842), mouse monoclonal Ob-R (leptin receptor) antibody (1:25 dilution; Santa Cruz, cat #sc-8391), mouse monoclonal adiponectin antibody (1:30 dilution; Abcam, cat #ab22554), rabbit monoclonal adiponectin receptor 1 antibody (1:350 dilution; Abcam, cat #ab126611), and goat polyclonal adiponectin receptor 2 antibody (1:25 dilution; Abcam, cat #ab77612). Optimal staining on control slides (human breast tissue TMAs) was obtained for each individual antibody. IHC was then performed using the optimized conditions on the experimental TMA slides constructed from WCHS specimens as well as on additional control slides. Primary antibodies were incubated at 37 °C for 1–2 h; secondary antibodies were incubated at 37 °C for 1 h, followed by either the DAB Map Detection Kit (Ventana, 760-124) or ChromoMap DAB kit (Ventana, 760-159). Slides were counterstained with hematoxylin (Ventana, 760-2021) and bluing reagent (Ventana, 760-2037) before cover slipping.

**Fig. 1:**
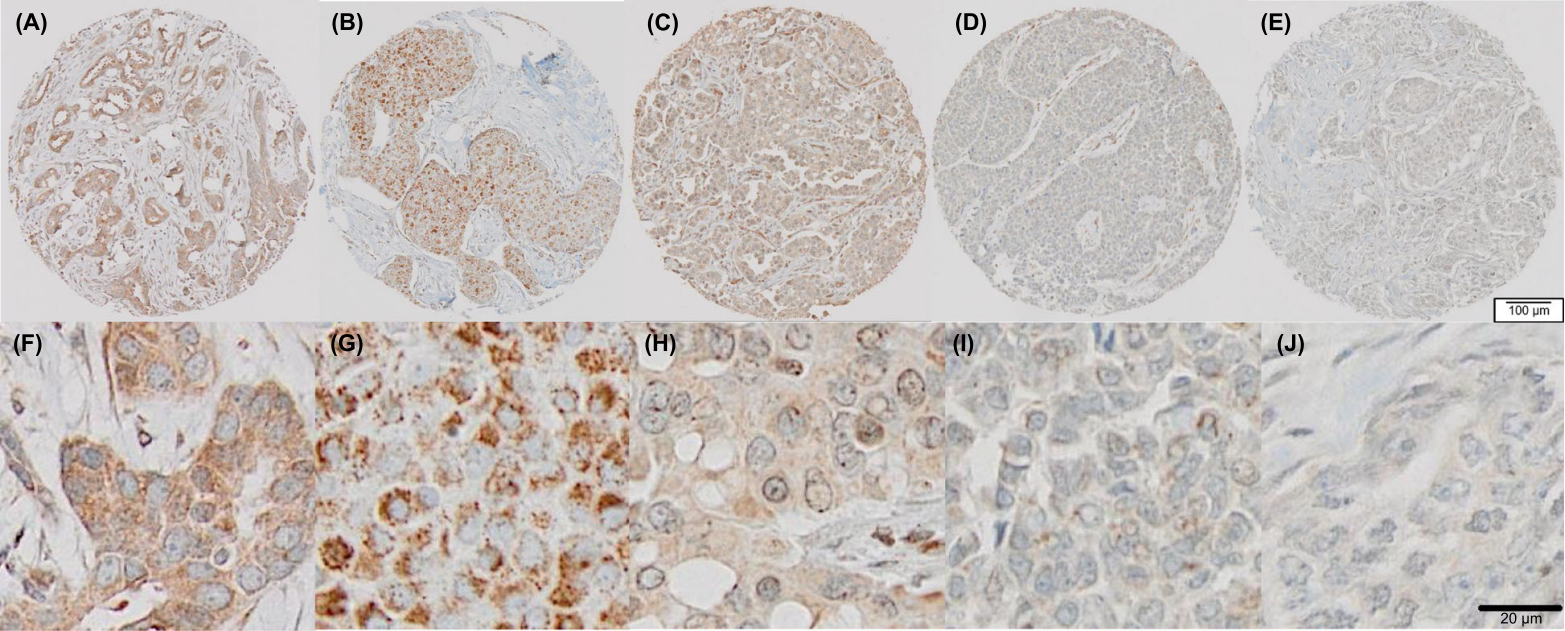
These images represent relatively average immunohistochemical expression of the adipokines and adipokine receptors in invasive breast tumor tissues. The specimens shown in **a** through **e** are imaged at × 20 magnification and show LEP (**a**), LEPR (**b**), ADIPOQ (**c**), ADIPOR1 (**d**), and ADIPOR2 (**e**) immunoreactivity in invasive breast tumor tissues. The images in **f** through **j** are further magnified images of each core shown in **a** through **e**. TMA cores were mostly 0.6 μm (with a range of 0.6 to 1.2 μm). Presence of granular cytoplasmic pattern of staining of the adipokines and adipokine receptors in the breast tumor tissues ranged in intensity from weakly positive (shown in images **d** and **e**—and the corresponding magnified images **i** and **j**) to moderate and strong (shown in images **a**–**c**—and the corresponding magnified images **f**–**h**)

TMA specimens stained for LEP, LEPR, ADIPOQ, ADIPOR1, and ADIPOR2 were digitized at × 20 on an Olympus VS120 whole slide scanner (Olympus Corporations, Central Valley, PA, USA). Examples of the resultant IHC staining for each biomarker on WCHS TMA specimens are shown in Fig. 1. TMA registration was performed on the software platform TMA-AID, as previously described [45], to correlate each study participant’s unique identification number with the corresponding imaged tissue cores and protein expression information. A digital pathology analysis platform (VisioPharm, Hoersholm Denmark) was used to build a custom workflow and perform quantitative analysis of IHC expression on each tissue core. Quantitative IHC expression results were reported as effective staining intensity (ESI) within the effective staining area (ESA) [45]. Specimen artifacts such as tissue folding were manually excluded from quantification. TMA-AID was also used by a board-certified pathologist (MC) to evaluate IHC expression for LEP and LEPR of each tissue core stained. Semi-quantitative expression results were reported as follows: 0 (negative), 1 (weak expression), 2 (moderate expression), or 3 (strong expression). We confirmed through Pearson’s correlation analysis that there were relatively high concordance rates between the automated/unsupervised (quantitative) and pathologist-generated (semi-quantitative) scores for LEP (*r* = 0.70; *P <* 0.0001) and LEPR (*r* = 0.71, *P <* 0.0001); therefore, for all five biomarkers examined, only the quantitative data were included in the analysis.

### Statistical analysis

Select sociodemographics, breast tumor clinicopathologic characteristics, and IHC expression of LEP, LEPR, ADIPOQ, ADIPOR1, and ADIPOR2 were described overall and by race using means (± standard deviations [SDs]) and frequencies (proportions) for continuous and categorical variables, respectively. Student’s *t* tests and analysis of variance (ANOVA) were used to test IHC expression of each biomarker by select characteristics. Pearson’s correlation analysis was used to assess pairwise correlations of adipokine and adipokine receptor IHC expression, overall and separately by race. Multivariate mixed effects models were used to describe the associations of breast tumor clinicopathologic features with adipokine and adipokine receptor IHC expression in order to incorporate the correlation among the adipokine and adipokine receptor IHC expression. Differences of least squares means were generated using multivariate mixed effects models controlling for race, BMI, and menopausal status. We decided a priori (based on background knowledge and a review of the literature) to control for these factors in the multivariate mixed effects models. In addition, each model was mutually controlled for the IHC expression of the other four biomarkers. Subsequent analysis focused on assessing the multivariate associations (controlling for BMI and menopausal status) between adipokine and adipokine receptor IHC expression with breast tumor clinicopathologic features among Black WCHS participants only.

Analyses were performed using SAS (v9.4 SAS Institute, Cary, NC). All reported *P* values are two-sided, and *P* < 0.05 was considered statistically significant. To address concerns of multiple comparisons in the multivariate mixed effects models assessing associations between adipokine IHC expression and tumor features, the Bonferroni correction was used, with a criterion for statistical significance of *P* < 0.003 (i.e., 0.05/15).

## Results

### Select characteristics of the study sample

Select characteristics of the WCHS participants included in the analysis are shown in Table 1, overall and with comparisons by race. The proportion of Black women in the sample is larger than the proportion of White women (540 Black vs. 159 White) because the study stopped recruiting White breast cancer cases in 2012, while enrollment of new Black breast cancer cases continued (through 2020). The current analysis included White cases diagnosed in 2001 through 2009 and Black cases diagnosed in 2001 through 2015, who had tumor specimens in the TMAs constructed by the start of the IHC analysis. Mean age at diagnosis was 52.6 ± 10.8 years, more than half of the sample was postmenopausal, and mean BMI was 30.7 ± 7.0 kg/m^2^. Several differences were observed by race: larger proportions of Black women were overweight or obese and had breast tumor features indicative of more aggressive phenotypes (e.g., tumors that were poorly differentiated, ER−, TN subtype) compared to White women.

**Table 1:** Select characteristics of the study sample, overall and by race

Sociodemographic and clinical characteristics	Overall (*N* = 720), *n* (%)	Black (*n* = 540), *n* (%)	White (*n* = 159), *n* (%)	*P* value
Age at diagnosis (years), mean ± SD	52.6 ± 10.8	52.6 ± 11.0	52.3 ± 10.1	0.75
Age at diagnosis (years)				0.46
20–45	200 (32.3)	148 (31.1)	52 (35.9)	
45–59	231 (37.3)	177 (37.3)	54 (37.2)	
≥ 60	189 (30.5)	150 (31.6)	39 (26.9)	
Menopausal status				0.07
Premenopausal	325 (46.5)	241 (44.6)	84 (52.8)	
Postmenopausal	374 (53.5)	299 (55.4)	75 (47.2)	
Race				
Black/African American	540 (77.2)	–	–	
White	159 (22.8)	–	–	
Body mass index (kg/m^2^), mean ± SD	30.7 ± 7.0	31.6 ± 6.9	27.6 ± 6.5	**< 0.0001**
Body mass index (kg/m^2^)				**< 0.0001**
18.5–24.99	177 (24.6)	88 (16.3)	68 (42.8)	
25.0–29.99	201 (27.9)	160 (29.6)	41 (25.8)	
30.0–34.99	176 (24.4)	147 (27.2)	29 (18.2)	
≥ 35.0	166 (23.1)	145 (26.9)	21 (13.2)	
Breast tumor clinicopathologic features				
Tumor grade				**< 0.0001**
Well differentiated	107 (16.9)	66 (14.1)	33 (22.6)	
Moderately differentiated	218 (34.4)	148 (31.7)	65 (44.5)	
Poorly differentiated	309 (48.7)	253 (54.2)	48 (32.9)	
Tumor size				0.36
< 1.0 cm	147 (20.4)	113 (20.9)	33 (20.8)	
1.0–2.0 cm	281 (39.0)	203 (37.6)	69 (43.4)	
> 2.0 cm	292 (40.6)	224 (41.5)	57 (35.8)	
AJCC stage				0.07
Stage 0	61 (8.8)	52 (10.1)	9 (5.7)	
Stage I	256 (37.1)	177 (34.5)	70 (44.6)	
Stage II	271 (39.3)	204 (39.8)	60 (38.2)	
Stage III	96 (13.9)	77 (15.0)	16 (10.2)	
Stage IV	6 (0.9)	3 (0.6)	2 (1.3)	
Lymph node status				0.38
Negative	406 (60.4)	298 (59.7)	98 (63.6)	
Positive	266 (39.6)	201 (40.3)	56 (36.4)	
Ki67 status^a^				0.26
Ki67−/favorable	348 (69.2)	272 (68.2)	69 (74.2)	
Ki67+/unfavorable	155 (30.8)	127 (31.8)	24 (25.8)	
ER status				**0.01**
ER−	215 (29.9)	175 (32.5)	35 (22.0)	
ER+	503 (70.1)	363 (67.5)	124 (78.0)	
PR status				**0.03**
PR−	333 (46.3)	260 (48.2)	64 (40.3)	
PR+	386 (53.7)	279 (51.8)	95 (59.7)	
HER2 status				0.59
HER2−	573 (81.5)	429 (81.6)	125 (79.6)	
HER2+	133 (18.5)	97 (18.4)	32 (20.4)	
Breast cancer subtype^b^				**0.03**
Luminal A	420 (59.8)	300 (57.1)	104 (66.2)	
Luminal B	60 (8.6)	46 (8.8)	14 (8.9)	
HER2-E	70 (10.0)	51 (9.7)	18 (11.5)	
TN	152 (21.6)	128 (24.4)	21 (13.4)	

Race was missing or unknown among 21 (2.9%) participants

^a^Ki67 status was missing or unknown among 217 (30.1%) participants

^b^Breast cancer subtypes were classified based on IHC expression of ER and PR, and overexpression or amplification of HER2 (by IHC or FISH) as reported in pathology records

### Summary of adipokine and adipokine receptor IHC expression in WCHS

Distributions of IHC expression of each biomarker by select characteristics are shown in Table 2. Some notable findings were that LEPR IHC expression was higher among postmenopausal women compared to premenopausal women and increased with increasing BMI. We also found that IHC expression of LEPR, ADIPOQ, and ADIPOR2 was significantly higher among Black women than White women, which was likely attributable to the finding that Black women were more frequently postmenopausal and overweight or obese. Variation in LEPR IHC expression was observed by breast tumor clinicopathologic features, while the expression of LEP, ADIPOQ, ADIPOR1, and ADIPOR2 did not appear to vary by tumor clinicopathology. Among Black women, older age, postmenopausal status, and increasing BMI were associated with higher LEPR IHC expression, and higher LEPR IHC expression was associated with Ki67−/favorable status, ER+ status, PR− status, and luminal A and luminal B subtypes (see Additional file 1). Among White women, only increasing BMI was associated with higher LEPR IHC expression, and higher LEPR IHC expression was associated with larger tumor size, positive lymph node status, and ER− status (see Additional file 2).

**Table 2:** Mean adipokine and adipokine receptor IHC expression, by select factors

Sociodemographic and clinical characteristics	LEP			LEPR			ADIPOQ			ADIPOR1			ADIPOR2		
	*n*	mean ± SD	*P*	*n*	mean ± SD	*P*	*n*	mean ± SD	*P*	*n*	mean ± SD	*P*	*n*	mean ± SD	*P*
Age at diagnosis (years)			0.53			0.05			0.69			0.11			0.45
20–45	181	112.2 ± 23.8		180	61.9 ± 26.2		200	73.4 ± 37.1		200	94.4 ± 38.5		200	61.8 ± 34.7	
45–59	215	111.8 ± 24.2		208	61.2 ± 26.8		231	74.6 ± 35.7		231	95.5 ± 37.6		231	59.0 ± 35.8	
60–75	179	109.4 ± 29.3		175	67.4 ± 27.8		189	76.5 ± 34.5		189	101.6 ± 32.6		189	63.2 ± 32.9	
Menopausal status			0.41			**0.01**			0.54			0.54			0.45
Premenopausal	300	111.8 ± 23.4		301	60.2 ± 27.2		325	74.0 ± 35.8		325	95.7 ± 37.5		325	60.0 ± 35.8	
Postmenopausal	350	110.2 ± 27.5		339	65.6 ± 26.3		374	75.6 ± 35.2		374	97.4 ± 35.2		374	62.0 ± 34.3	
Race			0.51			**< 0.0001**			**0.0003**			0.68			**0.03**
Black/African American	500	110.6 ± 24.0		495	66.3 ± 24.8		540	77.5 ± 34.2		540	97.0 ± 35.8		540	62.7 ± 34.4	
White	150	112.1 ± 30.6		145	52.1 ± 30.4		159	65.8 ± 38.3		159	95.6 ± 38.1		159	55.7 ± 36.6	
Body mass index (kg/m^2^)			0.39			**< 0.0001**			0.36			0.17			0.09
18.5–24.99	163	112.9 ± 25.1		160	55.8 ± 27.6		177	71.0 ± 38.4		177	92.3 ± 40.3		177	54.9 ± 36.2	
25.0–29.99	184	108.4 ± 25.1		182	58.9 ± 29.2		201	75.3 ± 33.9		201	98.5 ± 37.0		201	60.5 ± 34.4	
30.0–34.99	166	112.0 ± 25.7		161	65.4 ± 26.8		176	77.6 ± 33.8		176	99.8 ± 33.3		176	64.1 ± 35.6	
≥ 35.0	156	111.1 ± 26.3		154	71.7 ± 19.9		166	74.0 ± 36.2		166	94.1 ± 35.2		166	61.9 ± 34.4	
Breast tumor clinicopathologic features															
Tumor grade			0.63			0.27			0.84			0.65			0.07
Well differentiated	99	108.6 ± 29.9		94	65.4 ± 28.4		107	74.2 ± 36.6		107	95.8 ± 42.0		107	56.5 ± 37.5	
Moderately differentiated	203	111.3 ± 24.6		199	64.2 ± 27.2		218	72.9 ± 35.9		218	94.9 ± 36.2		218	57.2 ± 35.3	
Poorly differentiated	298	111.2 ± 24.8		291	61.1 ± 25.1		309	74.8 ± 35.7		309	63.3 ± 33.6		309	63.3 ± 33.6	
Tumor size			0.48			0.08			**0.03**			0.22			**0.003**
< 1.0 cm	124	112.4 ± 23.3		129	58.5 ± 33.0		147	81.0 ± 33.4		147	101.0 ± 42.8		147	60.7 ± 36.0	
1.0–2.0 cm	265	111.9 ± 25.8		256	65.0 ± 26.5		281	71.5 ± 36.8		281	95.2 ± 36.7		281	55.1 ± 36.1	
> 2.0 cm	280	109.6 ± 26.2		272	62.6 ± 23.8		292	74.1 ± 35.1		292	95.0 ± 32.9		292	65.1 ± 33.4	
AJCC stage			0.56			0.40			0.44			0.18			0.24
Stage 0	62	111.1 ± 27.7		51	67.3 ± 28.7		61	78.9 ± 34.5		61	91.3 ± 47.4		61	62.7 ± 37.7	
Stage I	241	112.1 ± 24.7		237	61.1 ± 29.8		256	75.1 ± 35.0		256	100.9 ± 35.6		256	57.0 ± 35.1	
Stage II	256	111.3 ± 23.1		246	64.0 ± 23.6		271	73.9 ± 35.5		271	94.1 ± 35.2		271	61.4 ± 35.5	
Stage III	91	106.9 ± 32.7		89	60.1 ± 25.3		96	69.8 ± 37.3		96	95.2 ± 34.2		96	65.1 ± 32.4	
Stage IV	6	106.8 ± 25.8		6	55.6 ± 28.6		6	88.6 ± 10.7		6	98.8 ± 10.0		6	45.6 ± 41.7	
Lymph node status			0.61			0.30			0.89			0.77			0.07
Negative	372	110.5 ± 26.5		368	61.8 ± 28.6		406	74.1 ± 35.4		406	96.7 ± 37.0		406	59.0 ± 35.2	
Positive	252	111.5 ± 25.4		244	64.2 ± 24.0		266	74.4 ± 36.1		266	95.8 ± 35.5		266	64.0 ± 34.4	
Ki67 status			0.09			0.12			0.05			0.34			0.64
Ki67−/favorable	326	110.0 ± 23.9		320	68.1 ± 25.5		348	79.0 ± 34.3		348	100.4 ± 37.1		348	61.8 ± 33.8	
Ki67+/unfavorable	147	113.8 ± 19.0		143	64.4 ± 22.4		155	72.6 ± 35.4		155	97.1 ± 32.9		155	63.3 ± 35.8	
ER status			0.45			**< 0.0001**			0.61			0.49			0.17
ER−	205	110.1 ± 23.8		198	55.4 ± 27.2		215	75.7 ± 34.3		215	97.9 ± 33.1		215	63.2 ± 34.1	
ER+	463	111.7 ± 25.8		459	65.9 ± 26.2		503	74.3 ± 35.9		503	96.0 ± 37.6		503	59.3 ± 35.6	
PR status			0.36			**< 0.0001**			0.57			0.46			**0.04**
PR−	307	110.2 ± 26.4		302	57.9 ± 27.0		333	73.8 ± 36.0		333	95.3 ± 36.3		333	63.2 ± 34.0	
PR+	361	112.0 ± 24.1		355	66.8 ± 26.2		386	75.3 ± 35.1		386	97.4 ± 36.6		386	57.9 ± 36.1	
HER2 status			0.15			0.24			0.35			0.37			0.42
HER2−	538	110.6 ± 25.6		521	63.4 ± 26.5		573	74.2 ± 36.3		573	96.1 ± 36.1		573	60.0 ± 35.0	
HER2+	121	114.2 ± 20.1		124	60.2 ± 27.4		130	77.2 ± 31.9		130	99.3 ± 36.3		130	62.8 ± 35.6	
Breast cancer subtype^a^			0.24			**< 0.0001**			0.71			0.58			0.12
Luminal A	393	110.6 ± 26.5		383	66.2 ± 26.1		420	73.9 ± 36.6		420	95.3 ± 37.2		420	58.5 ± 35.7	
Luminal B	55	117.7 ± 13.2		56	66.3 ± 26.1		60	79.3 ± 28.4		60	98.8 ± 35.7		60	58.3 ± 35.5	
HER2-E	66	111.2 ± 34.1		68	55.3 ± 27.7		70	75.4 ± 34.7		70	99.7 ± 37.1		70	66.5 ± 35.5	
TN	145	110.6 ± 23.3		138	55.8 ± 26.3		152	75.7 ± 35.3		152	99.1 ± 31.9		152	64.6 ± 32.7	

IHC expression scores reflect quantitative expression of LEP, LEPR, ADIPOQ, ADIPOR1, and ADIPOR2 as analyzed through an automated/unsupervised scoring (quantitative) methodology. The scores estimate the effective staining intensity (ESI) within the effective staining area (ESA) of the biomarker in question

^a^Breast cancer subtypes were classified based on IHC expression of ER and PR, and overexpression or amplification of HER2 (by IHC or FISH) as reported in pathology records

**Fig. 2:**
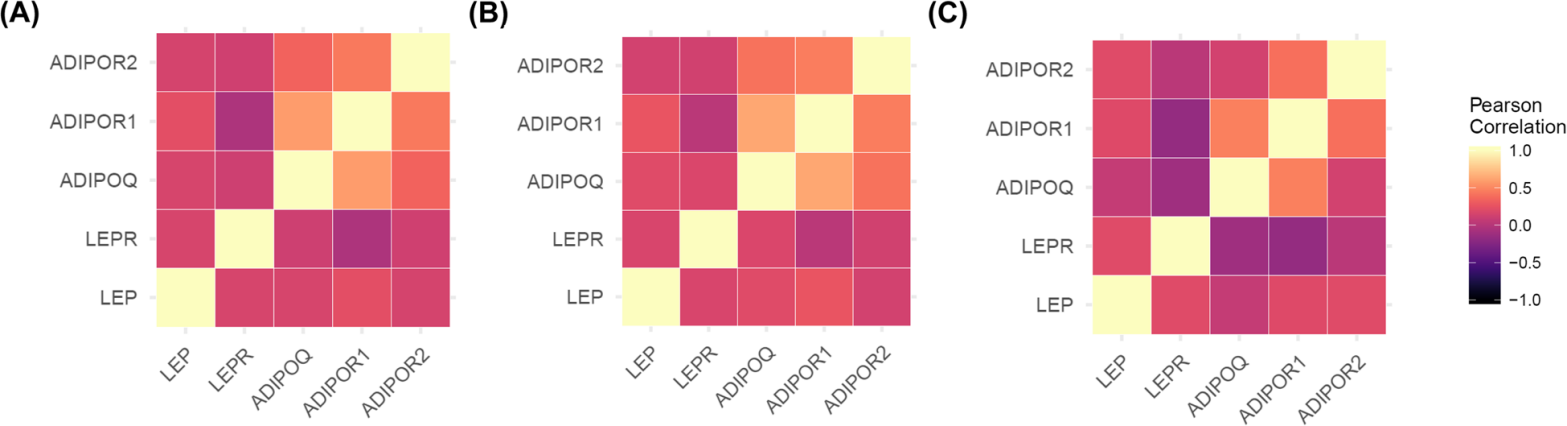
The pairwise Pearson correlations between IHC expression of LEP, LEPR, ADIPOQ, ADIPOR1, and ADIPOR2 among the overall study sample (*N* = 720) (**a**), among Black study participants only (*n* = 540) (**b**), and among White study participants only (*n* = 159) (**c**)

Figure 2 depicts the pairwise Pearson correlation matrix for IHC expression of LEP, LEPR, ADIPOQ, ADIPOR1, and ADIPOR2 in the overall sample (Fig. 2a), and separately among Black (Fig. 2b) and White study participants (Fig. 2c). There were significant positive correlations, although weak (*r* < 0.25), for LEP IHC expression with LEPR, ADIPOQ, ADIPOR1, and ADIPOR2 IHC expression. There was a moderate positive correlation for ADIPOQ with ADIPOR1 (*r* = 0.59) and a weak-to-moderate positive correlation for ADIOPQ with ADIPOR2 (*r* = 0.34). IHC expression of ADIPOR1 and ADIPOR2 were also moderately correlated (*r* = 0.44). It is worth noting that IHC expression of LEPR and ADIPOR1 were not significantly correlated. These findings were generally consistent in the subgroup analyses by race.

### Associations of adipokine and adipokine receptor IHC expression with breast cancer clinicopathology in WCHS

Table 3 shows the mixed effects models of the associations of LEP, LEPR, ADIPOQ, ADIPOR1, and ADIPOR2 IHC expression with breast tumor clinicopathologic features. LEP IHC expression was not significantly associated with any tumor clinicopathologic feature apart from breast cancer subtype. Higher LEP IHC expression was marginally associated with luminal B subtype (vs. luminal A, *P =* 0.05). Lower LEPR IHC expression was associated with ER− and PR− status, and HER2-E and TN subtypes. Lower ADIPOQ IHC expression was associated with larger tumor size and unfavorable Ki67 status. No statistically significant associations were observed between any tumor clinicopathologic feature and ADIPOR1 or ADIPOR2 IHC expression. After the Bonferroni correction (*P* < 0.003), only the observed associations of lower LEPR IHC expression with ER− status and TN subtype remained statistically significant.

**Table 3:** Multivariate mixed effects models of the associations of adipokine and adipokine receptor IHC expression with breast tumor clinicopathologic features in the overall study sample

Breast tumor clinicopathologic features	LEP		LEPR		ADIPOQ		ADIPOR1		ADIPOR2	
	Diff of LS means (SE)	*P*	Diff of LS means (SE)	*P*	Diff of LS means (SE)	*P*	Diff of LS means (SE)	*P*	Diff of LS means (SE)	*P*
Tumor grade										
Moderately vs. well differentiated	2.44 (3.29)	0.46	− 1.25 (3.25)	0.70	− 2.61 (4.31)	0.54	− 0.49 (4.26))	0.91	− 2.63 (4.19)	0.53
Poorly vs. well differentiated	2.88 (3.16)	0.36	− 5.52 (3.12)	0.08	− 1.97 (4.17)	0.64	1.92 (4.11)	0.64	2.58 (4.05)	0.53
Tumor size										
1.0–2.0 cm vs. < 1.0 cm	0.70 (2.84)	0.80	**6.25 (2.81)**	**0.03**	**− 9.72 (3.60)**	**0.007**	− 6.50 (3.72)	0.08	− 5.62 (3.57)	0.12
> 2.0 cm vs. < 1.0 cm	− 1.12 (2.83)	0.69	3.32 (2.79)	0.24	**− 7.86 (3.60)**	**0.03**	− 6.49 (3.71)	0.08	4.11 (3.56)	0.25
AJCC stage										
Stage I vs. stage 0	2.80 (4.11)	0.50	− 3.02 (3.99)	0.45	− 2.95 (5.03)	0.56	8.95 (5.18)	0.08	− 4.61 (5.03)	0.36
Stage II vs. stage 0	2.02 (4.08)	0.62	− 1.39 (3.96)	0.73	− 4.97 (4.99)	0.32	2.06 (5.13)	0.69	− 1.40 (4.99)	0.78
Stage III/IV vs. stage 0	− 2.90 (4.60)	0.53	− 5.93 (4.50)	0.19	− 9.02 (5.71)	0.11	2.30 (5.87)	0.69	1.26 (5.71)	0.83
Lymph node status										
Positive vs. negative	1.21 (2.20)	0.58	1.13 (2.16)	0.60	0.63 (2.83)	0.83	0.10 (2.89)	0.97	4.65 (2.78)	0.09
Ki67 status										
Ki67+/unfavorable vs. Ki67−/favorable	3.25 (2.29)	0.16	− 4.00 (2.35)	0.09	**− 7.16 (3.28)**	**0.03**	− 3.96 (3.47)	0.26	0.81 (3.30)	0.81
ER status										
ER− vs. ER+	− 1.17 (2.20)	0.59	**− 10.47 (2.20)**	**< 0.0001**	0.14 (2.91)	0.96	1.91 (3.00)	0.52	2.97 (2.89)	0.31
PR status										
PR− vs. PR+	− 1.92 (2.03)	0.34	**− 9.54 (2.02)**	**< 0.0001**	− 1.59 (2.67)	0.55	− 1.14 (2.75)	0.68	4.98 (2.65)	0.06
HER2 status										
HER2+ vs. HER2−	3.57 (2.55)	0.16	− 1.42 (2.60)	0.58	2.79 (3.43)	0.42	2.70 (3.50)	0.44	2.08 (3.41)	0.54
Breast cancer subtype^a^										
Luminal B vs. luminal A	**7.22 (3.65)**	**0.05**	1.64 (3.66)	0.65	4.65 (4.89)	0.34	3.41 (4.48)	0.49	− 0.61 (4.83)	0.90
HER2-E vs. luminal A	0.59 (3.37)	0.86	**− 9.46 (3.37)**	**0.005**	1.21 (4.56)	0.79	3.90 (4.65)	0.40	7.09 (4.52)	0.12
TN vs. luminal A	0.13 (2.50)	0.96	**− 10.92 (2.57)**	**< 0.0001**	0.04 (3.39)	0.99	3.54 (3.46)	0.31	5.24 (3.36)	0.12

Differences of least squares means were generated using multivariate mixed effects models controlling for race, BMI, and menopausal status. Each model also mutually controlled for the other four biomarkers examined

^a^Breast cancer subtypes were classified based on IHC expression of ER and PR, and overexpression or amplification of HER2 (by IHC or FISH) as reported in pathology records

Subgroup analysis of the multivariate associations of LEP, LEPR, ADIPOQ, ADIPOR1, and ADIPOR2 IHC expression with breast tumor clinicopathologic features among Black women yielded similar findings (Table 4). Some notable qualitative differences from the associations observed in the overall sample (shown in Table 3) suggested that the association of lower LEPR IHC expression with poorly differentiated tumors (vs. well differentiated) and unfavorable Ki67 status (vs. favorable) was stronger in magnitude among Black women as compared to the overall sample, while the association of lower LEPR IHC expression with PR− status was attenuated among Blacks. Additionally, the association of lower ADIPOQ IHC expression with unfavorable Ki67 status was slightly stronger, while the association of ADIPOQ IHC expression with increasing tumor size was attenuated among Black women.

**Table 4:** Multivariate mixed effects models of the associations of adipokine and adipokine receptor IHC expression with breast tumor clinicopathologic features among Black study participants only

Breast tumor clinicopathologic features	LEP		LEPR		ADIPOQ		ADIPOR1		ADIPOR2	
	Diff of LS means (SE)	*P*	Diff of LS means (SE)	*P*	Diff of LS means (SE)	*P*	Diff of LS means (SE)	*P*	Diff of LS means (SE)	*P*
Tumor grade										
Moderately vs. well differentiated	**− 8.20 (3.68)**	**0.03**	− 4.99 (3.61)	0.17	− 6.85 (5.06)	0.18	− 4.42 (4.99)	0.38	− 5.95 (5.01)	0.24
Poorly vs. well differentiated	− 5.50 (3.44)	0.11	**− 9.03 (3.38)**	**0.008**	− 5.59 (4.76)	0.24	− 2.03 (4.69)	0.66	− 0.37 (4.71)	0.94
Tumor size										
1.0–2.0 cm vs. < 1.0 cm	− 0.84 (3.07)	0.78	1.99 (3.06)	0.52	− 7.80 (4.01)	0.05	− 6.58 (4.19)	0.12	− 6.37 (4.02)	0.11
> 2.0 cm vs. < 1.0 cm	0.05 (3.01)	0.99	− 2.04 (3.01)	0.50	− 6.28 (3.94)	0.11	− 3.07 (4.13)	0.46	3.29 (3.96)	0.41
AJCC stage										
Stage I vs. stage 0	7.38 (4.25)	0.08	− 0.01 (4.12)	0.99	1.43 (5.36)	0.79	10.34 (5.57)	0.06	2.12 (5.46)	0.70
Stage II vs. stage 0	5.17 (4.20)	0.22	− 2.67 (4.08)	0.51	− 2.89 (5.28)	0.58	4.91 (5.49)	0.37	3.09 (5.38)	0.57
Stage III/IV vs. stage 0	1.38 (4.73)	0.77	− 7.55 (4.65)	0.10	− 5.76 (6.06)	0.34	4.63 (6.30)	0.46	4.90 (6.18)	0.43
Lymph node status										
Positive vs. negative	− 0.79 (2.39)	0.74	1.52 (2.33)	0.51	− 0.21 (3.13)	0.95	− 1.15 (3.22)	0.72	− 4.35 (3.12)	0.16
Ki67 status										
Ki67+/unfavorable vs. Ki67−/favorable	1.76 (2.26)	0.44	**− 4.82 (2.34)**	**0.04**	**− 8.95 (3.44)**	**0.01**	− 4.49 (3.64)	0.22	0.53 (3.59)	0.88
ER status										
ER− vs. ER+	− 3.22 (2.27)	0.16	**− 9.98 (2.32)**	**< 0.0001**	− 2.21 (3.13)	0.48	0.98 (3.27)	0.76	1.46 (3.17)	0.64
PR status										
PR− vs. PR+	− 4.82 (3.33)	0.15	− 6.36 (4.14)	0.13	− 4.15 (6.40)	0.52	− 6.16 (5.23)	0.24	− 3.13 (5.65)	0.58
HER2 status										
HER2+ vs. HER2−	2.68 (2.71)	0.32	− 0.65 (2.84)	0.82	1.98 (3.83)	0.61	2.00 (3.95)	0.61	0.51 (3.86)	0.89
Breast cancer subtype^a^										
Luminal B vs. luminal A	7.04 (3.87)	0.07	0.94 (3.99)	0.81	3.83 (5.42)	0.48	− 0.87 (5.59)	0.88	− 6.63 (5.45)	0.22
HER2-E vs. luminal A	− 1.62 (3.61)	0.65	**− 7.98 (3.71)**	**0.03**	− 0.18 (5.15)	0.97	6.13 (5.31)	0.25	7.97 (5.18)	0.12
TN vs. luminal A	− 1.01 (2.55)	0.69	**− 10.57 (2.66)**	**< 0.0001**	− 0.88 (3.60)	0.81	2.70 (3.71)	0.47	1.88 (3.62)	0.60

Differences of least squares means were generated using multivariate mixed effects models controlling for BMI and menopausal status. Each model also mutually controlled for the other four biomarkers examined

^a^Breast cancer subtypes were classified based on IHC expression of ER and PR, and overexpression or amplification of HER2 (by IHC or FISH) as reported in pathology records

## Discussion

Given observed associations of increasing levels of adiposity with poorer breast cancer survival, we examined relationships between breast tumor IHC expression of adipokine biomarkers (LEP, LEPR, ADIPOQ, ADIPOR1, ADIPOR2) with tumor clinicopathological features that are associated with poorer prognosis. Our overarching hypothesis was that higher IHC expression of LEP, LEPR, ADIPOQ, ADIPOR1, and ADIPOR2 within the breast tumor microenvironment is associated with breast cancer aggressiveness, but we generally observed the opposite. While we found that Black women, postmenopausal women, and those with BMI > 30.0 kg/m^2^ had significantly higher LEPR IHC expression in their breast tumors, these factors were not significantly associated with IHC expression of LEP, ADIPOQ, ADIPOR1, or ADIPOR2. In multivariable models, which controlled for race, BMI, and menopausal status, we found that lower LEPR IHC was associated with ER− status and TN subtype, and these associations were particularly strong among Black women. These findings suggest that lower expression of LEPR (rather than higher expression as hypothesized) is an important indicator of more aggressive breast cancer, independent of race, BMI, and menopausal status, and might serve as an important biomarker associated with disparate outcomes, particularly among Black women. This seemingly counterintuitive observation is partially supported by data suggesting complex associations of obesity/adiposity, LEP/LEPR’s activation of various signaling pathways, and breast cancer progression, which is further complicated by ER expression [46, 47]. We hypothesize that central adiposity (rather than general obesity, as measured by BMI) is the etiologic mechanism linking LEPR expression in the breast tumor microenvironment with aggressive tumor clinicopathology and ultimately poorer prognosis. This might explain the lack of strong significant associations between BMI and IHC expression of the adipokine biomarkers examined in this study. While not the focus of the present analysis, our next steps will be to examine other anthropometric measures (e.g., waist circumference, hip circumference, waist-to-hip ratio), as well as body composition measures (percent body fat), as predictors of adipokine expression in the tumor microenvironment, with consideration to differences by ER status.

While numerous epidemiologic studies have explored and confirmed significant associations between circulating levels of the adipokines with risk of obesity-related cancers [48], fewer studies have examined the associations of IHC expression of the adipokines and their receptors with breast cancer clinicopathology [49–57], and most studies to date have been conducted in predominantly non-Black study samples. Findings from some of these studies suggest that LEPR expression might be downregulated in invasive tumors [50, 55] (and those with more aggressive features, including TN subtypes and unfavorable Ki67 status [52]) as compared to DCIS and normal-adjacent tissues. These observations indicate an association between LEPR downregulation and breast cancer invasion and progression. In contrast to our findings, several studies examining LEPR expression in breast cancer did not report significant associations of lower LEPR IHC expression with tumor features indicative of more aggressive phenotype [49, 51, 55, 56]. These inconsistencies could be due to differences in the analytical approach used (e.g., semi-quantitative, discrete vs. quantitative, continuous assessment of IHC expression, which directly influences the selection of statistical methodology), differences in the characteristics of the study participants examined (our analysis of adipokine IHC expression included the largest number of Black breast cancer cases to date), and/or a lack of consideration of the associations studied herein. For instance, our study explored the associations of interest using an unsupervised, digital analysis platform allowing for continuous, quantitative measurement of LEP, LEPR, ADIPOQ, ADIPOR1, and ADIPOR2 IHC expression, which are more objective. Conversely, other studies [49–51, 53, 55, 57] have examined IHC expression of these biomarkers using semi-quantitative methods, where discrete scores were assigned based on staining intensity (e.g., negative vs. positive, or none, mild, moderate, intense) and staining distribution (e.g., 0, < 10%; 1+, 10–50%; 2+, 50–80%; 3+, > 80%). These methods tend to yield more subjective results. Additionally, our analytic approach allowed us to control for important confounders (BMI, menopausal status, and race).

Similar to the observations reported herein, there is evidence that lower LEPR IHC is associated with clinicopathologic features indicative of tumor progression or aggressiveness in other obesity-related cancers [58–63]. A study of colorectal cancer showed that downregulation of LEPR IHC expression was associated with aggressive tumor features (namely late stage, high grade) as well as with shorter survival time [61]. Osorio and colleagues [60] showed that while there was no significant association of LEP IHC expression with prostate tumor aggressiveness, quantitative IHC expression of LEPR was significantly lower in prostate tumors exhibiting prognostic factors indicative of aggressive phenotype (namely, urethral margin involvement, surgical margin involvement, and seminal vesicle involvement). Studies of thyroid cancer [58, 59] have also shown that downregulation of LEPR was associated with increased risk of thyroid cancer recurrence and metastasis, particularly in the anaplastic thyroid cancer (ATC) subtype [59]. In endometrial cancer [62, 63], although LEP and LEPR were more highly expressed in endometrial tumor tissues than normal tissues, there was a suggestion that rates of LEPR positivity were significantly lower among poorly differentiated endometrial tumors. Notably, one study [62] showed that downregulation of the short form of LEPR was significantly associated with poor tumor differentiation in endometrial cancer. This finding indicates that there could be differences in the association of LEPR expression based on the isoform analyzed. Moreover, the complexity of the relationship of adiposity and deregulation of adipokine signaling with breast cancer (as mentioned above) might be similarly complex in other obesity/adiposity-related cancers [60, 64, 65], warranting additional research.

Our findings that lower ADIPOQ IHC expression was associated with ER− status and TN subtype, which although were not of statistical significance after the Bonferroni correction, are nonetheless worth noting. Several properties of ADIPOQ [11, 14, 27, 30, 31] imply a protective role in breast carcinogenesis. However, evidence suggests that there is a dichotomy observed in the relationship between ADIPOQ and breast cancer progression by ER status [66, 67]. In vitro studies have shown ADIPOQ stimulates growth in ER+ breast cancer cells and inhibits cellular proliferation in ER− breast cancer cells [66, 67]. So, lower IHC expression of ADIPOQ might be consistent with breast tumors progressing towards increasing aggressiveness (but only in some instances). The significant inverse associations of ADIPOQ IHC expression observed with larger tumor size and unfavorable Ki67 status also support this hypothesis. Additional research, particularly in large, diverse samples, is essential to elucidate the role of the ADIPOQ in carcinogenesis (along with the other adipokines and adipokine receptors examined herein), specifically in the tissue-specific microenvironment, which could contribute to the identification of new targets for obesity-related cancers. Moreover, as ADIPOQ exerts its effects through ADIPOR1 and ADIPOR2, more in-depth analysis of the predictors of ADIPOQ, ADIPOR1, and ADIPOR2 IHC expression in the tumor microenvironment and the impacts of their expression on tumor clinicopathology and subsequent survival outcomes, by adiposity type (e.g., overall adiposity, visceral) as well as by ER status, are critically needed.

A major strength of this study was the assessment of quantitative IHC expression and analysis of the associations of interest in a sample of breast cancer cases with well-defined clinicopathologic annotations, including > 500 Black breast cancer cases with TMA specimens and comprehensive clinicopathology data. Another strength was the relatively large number of samples included in the IHC analysis, which is one of, if not, the largest studies of adipokine and adipokine receptor IHC expression in breast cancer to date. There were also some limitations that should be considered. The first concern is the relatively small sample size of White study participants, which limited the power to detect meaningful differences in the associations of interest by race. The lack of complete and detailed clinical information on Ki67 expression was also a concern. Ki67 status was not available in the medical records of 217 participants (30.1% of the analytic sample). Unfortunately, we were also unable to retrieve the exact percentage of Ki67 staining in the medical records of those participants among whom the data were available, so we crudely classified Ki67 status as either clinically favorable (negative or borderline) or clinically unfavorable (positive) in the analysis. Despite these limitations, our findings support the hypothesis that IHC expression of the adipokines and adipokine receptors, particularly LEPR, is associated with tumor features that are indicative of more aggressive breast cancer phenotypes.

## Conclusions

In summary, findings from this study suggest that lower LEPR IHC expression within the breast tumor microenvironment might serve as an indicator of increased breast tumor aggressiveness. This study focused primarily on breast cancer clinicopathologic features as a first step in assessing the association of adipokine and adipokine receptor expression on breast cancer prognosis, as breast clinicopathologic features that might be indicative of more aggressive phenotypes are likely to affect survival. Additional studies are needed to clarify the clinical implications of LEPR expression and the mechanisms involved in the regulation of LEPR expression, to ultimately determine the utility of this biomarker in understanding breast tumor aggressiveness.

## Supplementary information

**Additional file 1: Table S1.** Mean adipokine and adipokine receptor IHC expression, by select factors, among Black participants only.

**Additional file 2: Table S2.** Mean adipokine and adipokine receptor IHC expression, by select factors, among White participants only.

## Data Availability

The datasets generated and analyzed during the current study are available from the corresponding author on reasonable request.

## Abbreviations

ADIPOQ: Adiponectin
ADIPOR1: Adiponectin receptor 1
ADIPOR2: Adiponectin receptor 2
AJCC: American Joint Committee on Cancer
ANOVA: Analysis of variance
BMI: Body mass index
DBBR: Data Bank and BioRepository
DCIS: Ductal carcinoma in situ
ER: Estrogen receptor
ESA: Effective staining area
ESI: Effective staining intensity
FFPE: Formalin-fixed paraffin-embedded
H&E: Hematoxylin and eosin
HER2: Human epidermal growth factor receptor 2
HER2-E: Non-luminal HER2-enriched subtype
IGF-1: Insulin-like growth factor 1
IHC: Immunohistochemistry
IRS1: Insulin receptor substrate 1
JAK/STAT: Janus kinase-signal transducer and activator of transcription
LEP: Leptin
LEPR: Leptin receptor
MAPK: Mitogen-activated protein kinase
PR: Progesterone receptor
SD: Standard deviation
SOCS3: Suppressor of cytokine signaling 3
TMA: Tissue microarray
TN: Triple-negative subtype
TNF-α: Tumor necrosis factor alpha
US: United States
WCHS: Women’s Circle of Health Study
