# Supplementary material for: Immunohistochemical analysis of adipokine and adipokine receptor expression in the breast tumor microenvironment: associations of lower leptin receptor expression with estrogen receptor-negative status and triple-negative subtype
Source: Breast Cancer Res. 2020 Feb 11;22:18. doi: 10.1186/s13058-020-1256-3 (PMC7014630; doi:10.1186/s13058-020-1256-3)
Supplement: Supplementary file 1 — Additional file 1: Table S1. Mean adipokine and adipokine receptor IHC expression, by select factors, among Black participants only. [file 13058_2020_1256_MOESM1_ESM.pdf]

Supplementary Table 1. Mean adipokine and adipokine receptor IHC expression[a], by select factors, among Black participants only

Sociodemographic and clinical characteristics	LEP			LEPR			ADIPOQ			ADIPOR1			ADIPOR2		
	n	mean±SD	P	n	mean±SD	P	n	mean±SD	P	n	mean±SD	P	n	mean±SD	P
Age at diagnosis (years)			0.54			**0.03**			0.67			0.09			0.90
20-45	134	109.4±25.0		134	64.0±25.5		148	76.0±35.9		148	94.9±39.1		148	62.1±34.7	
45-59	163	112.3±21.2		159	65.1±23.6		177	77.2±34.5		177	94.1±36.7		177	62.4±34.3	
60-75	141	109.9±27.0		139	71.2±25.1		150	79.5±33.6		150	102.3±32.6		150	63.7±33.5	
Menopausal status			0.37			**0.02**			0.58			0.85			0.89
Premenopausal	223	110.2±23.3		225	63.3±25.5		241	76.6±34.4		241	97.3±36.0		241	62.5±34.8	
Postmenopausal	277	110.9±24.7		270	68.8±24.1		299	78.2±34.1		299	96.7±35.7		299	62.9±34.1	
Body mass index (kg/m^2)			0.68			**0.002**			0.52			0.28			0.95
18.5-24.99	79	108.0±27.2		81	60.9±25.9		88	72.8±39.1		88	92.8±41.0		88	60.8±33.3	
25.0-29.99	147	110.0±22.2		145	62.6±27.0		160	78.4±32.1		160	100.3±35.6		160	62.8±34.9	
30.0-34.99	138	111.7±24.7		134	67.5±26.0		147	79.4±33.3		147	98.7±33.2		147	63.1±36.7	
≥35.0	136	111.5±23.4		135	72.2±18.8		145	77.4±34.1		145	94.1±34.9		145	63.4±33.4	
Breast tumor clinicopathologic features															
Tumor grade			0.12			**0.01**			0.38			0.63			0.24
Well differentiated	63	115.8±15.4		61	73.5±22.2		66	82.8±30.6		66	99.7±33.9		66	65.3±34.2	
Moderately differentiated	136	108.4±24.4		136	68.5±25.7		148	75.9±35.5		148	95.4±35.9		148	59.3±34.5	
Poorly differentiated	244	110.2±25.3		237	63.9±22.7		253	77.1±34.1		253	98.0±32.1		253	64.9±33.2	
Tumor size			0.76			0.28			0.21			0.35			**0.01**
<1.0 cm	94	112.2±21.2		98	66.2±28.7		113	82.4±33.6		113	100.0±43.4		113	63.2±35.7	
1.0-2.0 cm	190	110.1±24.3		188	68.4±25.5		203	75.4±35.4		203	94.3±36.0		203	57.4±34.8	
>2.0 cm	216	110.2±25.0		209	64.4±22.1		224	77.0±33.3		224	97.8±31.0		224	67.2±32.8	
AJCC stage			0.37			0.24			0.44			0.22			0.86
Stage 0	40	107.8±27.8		43	69.6±28.1		52	77.7±36.4		52	89.5±49.4		52	58.9±38.4	
Stage I	168	112.6±21.5		167	68.1±25.9		177	80.8±31.4		177	101.8±32.9		177	62.1±34.1	
Stage II	192	110.7±20.7		187	65.9±22.2		204	76.4±33.9		204	96.2±33.7		204	63.2±33.9	
Stage III	73	106.2±33.4		70	61.6±24.1		77	72.8±37.2		77	95.9±33.5		77	65.5±33.3	
Stage IV	3	114.8±12.9		3	50.7±27.0		3	89.8±1.6		3	101.8±6.4		3	56.5±49.2	
Lymph node status			0.91			0.59			0.86			0.67			0.12
Negative	271	110.1±23.9		269	67.0±25.4		298	77.4±34.5		298	96.5±36.6		298	61.5±35.3	
Positive	191	110.3±25.9		188	65.8±23.5		201	77.9±33.4		201	97.9±32.9		201	66.2±31.8	
Ki67 status			0.40			**0.03**			**0.01**			0.23			0.12
Ki67-/favorable	257	110.6±21.2		255	72.2±21.8		272	83.9±31.1		272	102.0±34.6		272	65.1±32.8	
Ki67+/unfavorable	120	112.5±18.2		118	67.0±20.5		127	75.0±33.6		127	97.6±32.2		127	65.6±34.4	
ER status			0.11			**<0.0001**			0.45			0.75			0.68
ER-	165	108.4±23.5		161	59.0±25.2		175	76.2±34.4		175	98.0±34.5		175	63.8±32.9	
ER+	334	112.0±23.5		334	69.8±23.9		363	78.6±33.7		363	97.0±35.8		363	62.5±34.9	
PR status			0.21			**<0.0001**			0.36			0.49			0.17
PR-	240	112.1±21.6		237	71.4±23.4		260	78.9±33.2		260	98.1±35.0		260	60.9±35.8	
PR+	259	109.4±25.4		258	60.8±25.3		279	76.2±35.0		279	96.0±36.2		279	64.9±32.5	
HER2 status			0.39			0.53			0.57			0.54			0.93
HER2-	401	110.6±23.6		394	66.7±24.6		429	77.7±34.9		429	97.2±35.0		429	63.2±34.1	
HER2+	91	112.9±20.4		91	64.9±24.7		97	79.7±29.6		97	99.6±35.6		97	63.5±35.0	
Breast cancer subtype[b]			0.20			**<0.0001**			0.89			0.61			0.18
Luminal A	280	111.0±24.2		278	70.1±23.5		300	78.2±34.8		300	96.6±35.8		300	62.8±34.9	
Luminal B	42	117.8±12.2		42	69.0±25.6		46	81.7±24.9		46	96.0±36.5		46	55.6±36.6	
HER2-E	49	108.6±24.7		49	61.3±23.5		51	77.9±33.4		51	102.8±35.8		51	70.6±32.1	
TN	121	109.6±21.9		116	58.5±25.2		128	77.1±34.7		128	99.4±32.3		128	64.4±31.9	

[a] IHC expression scores reflect quantitative expression of LEP, LEPR, ADIPOQ, ADIPOR1, and ADIPOR2 as analyzed through an automated/unsupervised scoring (quantitative) methodology. The scores estimate the effective staining intensity (ESI) within the effective staining area (ESA) of the biomarker in question.
[b] Breast cancer subtypes were classified based on IHC expression of ER and PR, and overexpression or amplification of HER2 (by IHC or FISH) as reported in pathology records.
