# Supplementary material for: Immunohistochemical analysis of adipokine and adipokine receptor expression in the breast tumor microenvironment: associations of lower leptin receptor expression with estrogen receptor-negative status and triple-negative subtype
Source: Breast Cancer Res. 2020 Feb 11;22:18. doi: 10.1186/s13058-020-1256-3 (PMC7014630; doi:10.1186/s13058-020-1256-3)
Supplement: Supplementary file 2 — Additional file 2: Table S2. Mean adipokine and adipokine receptor IHC expression, by select factors, among White participants only. [file 13058_2020_1256_MOESM2_ESM.pdf]

Supplementary Table 2. Mean adipokine and adipokine receptor IHC expression[a], by select factors, among White participants only

Sociodemographic and clinical characteristics	**LEP**			**LEPR**			**ADIPOQ**			**ADIPOR1**			**ADIPOR2**		
	**n**	**mean±SD**	**P**	**n**	**mean±SD**	**P**	**n**	**mean±SD**	**P**	**n**	**mean±SD**	**P**	**n**	**mean±SD**	**P**
Age at diagnosis (years)			0.11			0.50			0.99			0.59			0.10
20-45	47	120.2±17.7		46	56.0±27.4		52	66.0±40.0		52	93.0±37.2		52	61.2±35.3	
45-59	52	110.5±32.1		49	48.5±32.3		54	66.0±38.5		54	100.1±40.3		54	48.0±38.6	
60-75	38	107.5±37.0		36	52.6±32.9		39	64.9±35.7		39	98.9±33.0		39	61.1±31.0	
Menopausal status			0.07			0.65			0.84			0.13			0.34
Premenopausal	77	116.6±23.5		76	51.0±30.0		84	66.4±38.9		84	91.3±41.3		84	53.1±37.9	
Postmenopausal	73	107.4±36.3		69	53.3±30.9		75	65.2±37.8		75	100.4±33.7		75	58.6±35.1	
Body mass index (kg/m^2)			0.07			**0.04**			0.17			0.46			0.18
18.5-24.99	65	118.3±22.9		62	50.7±29.1		68	71.1±37.7		68	94.1±37.7		68	53.6±38.4	
25.0-29.99	37	102.1±34.0		37	44.1±32.8		41	63.3±38.0		41	91.7±41.6		41	51.6±31.1	
30.0-34.99	28	113.5±30.9		27	54.9±29.1		29	68.3±35.5		29	105.7±33.7		29	69.1±35.2	
≥35.0	20	108.5±41.4		19	68.1±26.9		21	50.2±42.4		21	94.1±38.1		21	51.9±40.2	
Breast tumor clinicopathologic features															
Tumor grade			**0.002**			0.75			0.73			0.52			0.60
Well differentiated	29	93.1±45.3		26	48.9±35.6		33	59.7±42.8		33	88.3±53.0		33	49.1±37.8	
Moderately differentiated	62	116.9±24.9		59	54.1±28.9		65	65.9±35.8		65	95.6±37.1		65	53.5±37.3	
Poorly differentiated	47	115.4±23.8		48	51.0±29.9		48	65.7±41.1		48	98.1±28.4		48	57.4±34.9	
Tumor size			0.22			**0.0007**			0.12			**0.05**			0.29
<1.0 cm	30	113.0±29.4		30	33.7±34.8		33	77.9±32.5		33	105.1±41.5		33	54.1±35.8	
1.0-2.0 cm	66	116.3±29.9		61	56.0±27.7		69	61.6±39.7		69	98.7±37.0		69	51.5±39.1	
>2.0 cm	54	106.6±31.9		54	57.9±27.1		57	63.9±38.7		57	86.3±35.9		57	61.6±33.5	
AJCC stage			0.44			0.07			0.39			0.50			**0.05**
Stage 0	8	127.6±21.9		8	55.1±30.5		9	85.5±20.7		9	101.9±34.0		9	84.8±34.9	
Stage I	65	110.6±32.0		63	44.0±33.1		70	63.1±40.0		70	101.0±39.9		70	48.6±34.2	
Stage II	58	113.6±29.8		54	58.4±26.8		60	66.9±38.4		60	89.8±38.5		60	57.6±39.8	
Stage III	15	107.5±32.6		16	55.0±27.7		16	55.8±37.4		16	90.3±34.2		16	64.9±30.5	
Stage IV	2	89.4±45.6		2	80.9±3.9		2	80.5±16.8		2	90.8±14.5		2	52.1±36.6	
Lymph node status			0.48			**0.04**			0.83			0.48			0.39
Negative	92	111.5±33.3		90	48.1±32.5		98	64.6±37.4		98	97.4±38.1		98	54.6±33.4	
Positive	53	115.0±24.9		53	58.3±24.8		56	66.0±40.9		56	92.9±39.6		56	59.8±41.5	
Ki67 status			0.14			0.85			0.87			0.80			0.79
Ki67-/favorable	64	108.1±32.8		60	52.5±32.5		69	61.9±38.8		69	97.7±43.3		69	53.2±33.5	
Ki67+/unfavorable	23	119.0±23.1		22	53.9±26.4		24	63.5±41.1		24	95.2±37.4		24	55.5±39.9	
ER status			0.24			**0.02**			0.12			0.37			0.20
ER-	115	110.5±32.0		112	55.3±29.8		124	63.4±38.8		124	94.5±40.9		124	53.7±35.5	
ER+	35	117.5±25.3		35	41.1±30.4		35	74.5±35.7		35	99.6±25.5		35	62.6±39.9	
PR status			0.80			0.92			0.79			0.85			0.23
PR-	91	111.6±30.6		86	54.8±30.2		95	65.2±37.9		95	95.1±40.1		95	52.9±35.2	
PR+	59	112.9±31.0		59	48.1±30.5		64	66.8±39.1		64	96.3±35.1		64	59.9±38.4	
HER2 status			0.08			0.44			0.42			0.59			0.37
HER2-	120	110.2±32.3		112	53.1±30.4		125	64.2±38.5		125	94.3±37.5		125	53.7±35.6	
HER2+	29	118.3±19.2		32	48.3±31.0		32	70.3±38.0		32	98.3±39.4		32	60.1±38.5	
Breast cancer subtype[b]			0.49			0.08			0.80			0.51			0.06
Luminal A	99	109.0±32.5		92	55.7±30.4		104	63.3±38.5		104	93.3±39.0		104	50.3±34.8	
Luminal B	13	117.5±16.5		14	57.9±27.0		14	71.6±38.1		14	108.1±35.9		14	67.4±31.4	
HER2-E	16	118.9±21.7		18	40.8±32.6		18	69.2±39.1		18	90.7±41.2		18	54.4±43.2	
TN	21	115.3±31.3		21	41.1±28.1		21	68.6±39.0		21	99.0±29.6		21	70.7±35.8	

[a] IHC expression scores reflect quantitative expression of LEP, LEPR, ADIPOQ, ADIPOR1, and ADIPOR2 as analyzed through an automated/unsupervised scoring (quantitative) methodology. The scores estimate the effective staining intensity (ESI) within the effective staining area (ESA) of the biomarker in question.

[b] Breast cancer subtypes were classified based on IHC expression of ER and PR, and overexpression or amplification of HER2 (by IHC or FISH) as reported in pathology records.
